# The Master’s Tools Will Never Dismantle the Master’s House: The Role of Diversity in Anti-Racist Education

**DOI:** 10.1093/geroni/igab046.471

**Published:** 2021-12-17

**Authors:** Karen Lincoln

**Affiliations:** University of Southern California, Los Angeles, California, United States

## Abstract

Diversity is a strange fruit that requires critical analysis to understand its meaning, value and impact on education. Depending on the era, diversity has been defined in a number of different ways and has a variety of meanings across a range of contexts. The lack of shared meaning and understanding of diversity and who controls the diversity narrative have significant implications for the development of anti-racist pedagogy in gerontological education. This presentation will discuss the history and evolution of the “diversity discourse” and how mainstream notions of diversity impact diversity initiatives, curriculum design and anti-racist pedagogy. Strategies for engaging in an historical analysis of diversity and how this process relates to the design, leadership and ownership of anti-racist curriculum will be discussed, as well as the role of gerontology in leading these efforts.

